# Breakfast consumption frequency is associated with dyslipidemia: a retrospective cohort study of a working population

**DOI:** 10.1186/s12944-022-01641-x

**Published:** 2022-03-27

**Authors:** Qi-mei Li, Cheng-kai Wu, Peng-cheng Ma, Hao Cui, Rui-ning Li, Chang Hong, Lin Zeng, Sheng-wu Liao, Lu-shan Xiao, Li Liu, Wen-yuan Li

**Affiliations:** 1 Big Data Center, Nanfang Hospital, Southern Medical University, 510515 Guangzhou, China; 2 Department of Infectious Diseases, Nanfang Hospital, Southern Medical University, 510515 Guangzhou, China; 3 Hospital Office, Nanfang Hospital, Southern Medical University, 510515 Guangzhou, China

**Keywords:** Breakfast, Dyslipidemia, Hypertriglyceridaemia, Working population, Diet, Regression analysis

## Abstract

**Background:**

Dyslipidemia is a significant contributor to cardiovascular and cerebrovascular diseases. Research on the relationship between breakfast consumption frequency and dyslipidemia in the working population is lacking. Therefore, we aimed to investigate this relationship based on a retrospective cohort study of a large working population in China.

**Methods:**

This retrospective cohort study used data from the physical examinations and questionnaire survey of working participants at Nanfang Hospital from January 20, 2015 to October 16, 2020. Univariate and multivariate analyses were conducted to explore the relationship between breakfast consumption frequency and dyslipidemia in this working population (*n* = 7644).

**Results:**

The prevalence of dyslipidemia among the participants was 26.4%. The univariate logistic regression test showed that the breakfast consumption frequency was inversely correlated with dyslipidemia. After adjusting for multiple factors, such as sex, age, body mass index, hypertension, hyperuricaemia, diabetes, smoking status, alcohol consumption, education level, marital status, long-term exposure to kitchen oil fumes, attending business dinners, and sleep time, it was found that breakfast consumption remained inversely associated with dyslipidaemia. The odds ratio for daily breakfast consumption was 0.466 (95% confidence interval 0.283–0.770, *P* = 0.003). After adjusting for confounding factors, we found that the higher the frequency of breakfast consumption, the lower the odds ratios for hypertriglyceridaemia.

**Conclusions:**

This study demonstrated that breakfast consumption frequency was inversely correlated with dyslipidemia. The higher the frequency of breakfast, the lower the risk of hypertriglyceridaemia. This study provides a basis on which dietary suggestions for the working population and lifestyle guidance for patients with a clinical need to prevent dyslipidemia can be made.

**Supplementary Information:**

The online version contains supplementary material available at 10.1186/s12944-022-01641-x.

## Background

Dyslipidemia is the main risk factor for atherosclerotic cardiovascular disease [1]. A study showed the changes in per capita blood cholesterol content from 1980 to 2018, involving 102.6 million adults from more than 200 countries worldwide. Dyslipidemia has become a major public health problem in China. A study used data from the ChinaPatient-Centered Evaluative Assessment of Cardiac Events Million Persons Project shows that one third of 35-75-year-old community residents in China suffer from dyslipidemia [2].

Many epidemiological and observational studies have demonstrated a relationship between diet, lifestyle factors, and dyslipidemia [3–6]. According to several studies, irregular diet is directly related to several cardiometabolic health outcomes, including obesity, weight gain, dyslipidemia, hypertension, diabetes, and others [7, 8]. Many studies have investigated the relationship between eating habits and metabolic syndrome [9–11]. With social and economic development, the eating habits and lifestyles of Chinese individuals have undergone significant changes, especially those of working people. According to surveys, changes in eating habits are associated with the incidence of metabolic syndrome and contribute to the risk of cardiovascular and cerebrovascular diseases [12–14]. The working population comprises mostly young to middle-aged people. The influence of dietary habits on dyslipidemia may aggravate the risk of cardiovascular and cerebrovascular diseases, and the impact on socioeconomics cannot be ignored. It is generally believed that breakfast is the most important meal of the day. However, there have been studies on the increasing prevalence of breakfast skipping, such as that from the United States, which reported that the prevalence has increased over the past 50 years, with up to 23.8% of young people skipping breakfast daily [15, 16]. It has been reported that skipping breakfast is related to adolescent cardiometabolic risk factors such as an altered lipid profile [17]. Previous studies have found that metabolic syndrome is inversely related to breakfast consumption frequency [13, 18]. Weight gain is also associated with the consumption of breakfast [14, 19, 20]. However, research on the relationship between breakfast consumption frequency and dyslipidemia in the working population is lacking. Therefore, this study investigated this relationship based on a retrospective cohort study of a large working population in China.

## Methods

### Research population and data collection

This cohort was derived using the Guangdong Provincial Key Area R&D Program (2019B020227004) for large-scale cohort study and clinical sample bank and database construction. From 20 January to 2015 to 16 October 2020, a total of 8287 people underwent physical examinations and completed questionnaire surveys at Nanfang Hospital, and we retrospectively collected these data. Physical examination included measuring height, weight, and detection of serum lipids. Data on demographic information, educational level, marital status, medical history of hypertension and diabetes, frequency of eating breakfast, sleep time, exposure to kitchen oil fumes, and business dinner attendance were collected using questionnaires managed by well-trained interviewers. Breakfast consumption frequency was assessed by the question. “How often do you have breakfast?” Answers were categorized as “never”, “1–3 times per week”, “4–6 times per week”, or “everyday”. Participants who consume breakfast less than four times per week were defined as “breakfast skippers” [21]. Physical measurements, such as height and weight were performed according to standard protocols. Data on the participants’ fasting plasma levels were collected, and blood lipid as well as uric acid parameters were recorded. Blood lipid parameters comprised total cholesterol (TC), low density lipoprotein cholesterol (LDL-C), high density lipoprotein cholesterol (HDL-C), and triglyceride (TG). Participants aged ≥ 18 years old who underwent physical examinations and completed questionnaire surveys were included. Participants with missing data on variables such as height and weight, education level, sleep, diet, job type, blood lipid parameters, and blood pressure were excluded. The final study sample comprised 7644 participants (4440 men, 3204 women).

### Dyslipidemia assessment

 Dyslipidemia was determined according to the 2013 ACC/AHA guideline. High TC was defined as TC ≥ 6.2 mmol/L, high LDL-C as LDL-C ≥ 4.1 mmol/L, low HDL-C as HDL-C < 1.0 mmol/L, and high TG as TG ≥ 2.3 mmol/L [22].

### Statistical analyses

The participants were divided into two groups: those with dyslipidemia and those without dyslipidemia. An independent sample t-test, chi-square test, or Mann-Whitney U test was used to assess the correlation between dyslipidemia and categorical or continuous variables. Odds ratios (ORs) were used to evaluate the independent and combined effects of breakfast consumption frequency on the prevalence of dyslipidemia. A logistic regression model was used for univariate and multivariate analyses to determine the variables related to dyslipidemia. The ORs for dyslipidemia and the variables were calculated, with corresponding 95% confidence intervals (CIs). The variables were adjusted to compare the ORs for dyslipidemia and hypertriglyceridaemia between the different models. All tests were two-sided, and a *P* value < 0.05 was considered statistically significant. All statistical analyses were performed using SPSS version 26.0 software (IBM Corp., Armonk, NY, USA).

## Results

### Baseline characteristics

**Fig. 1:**
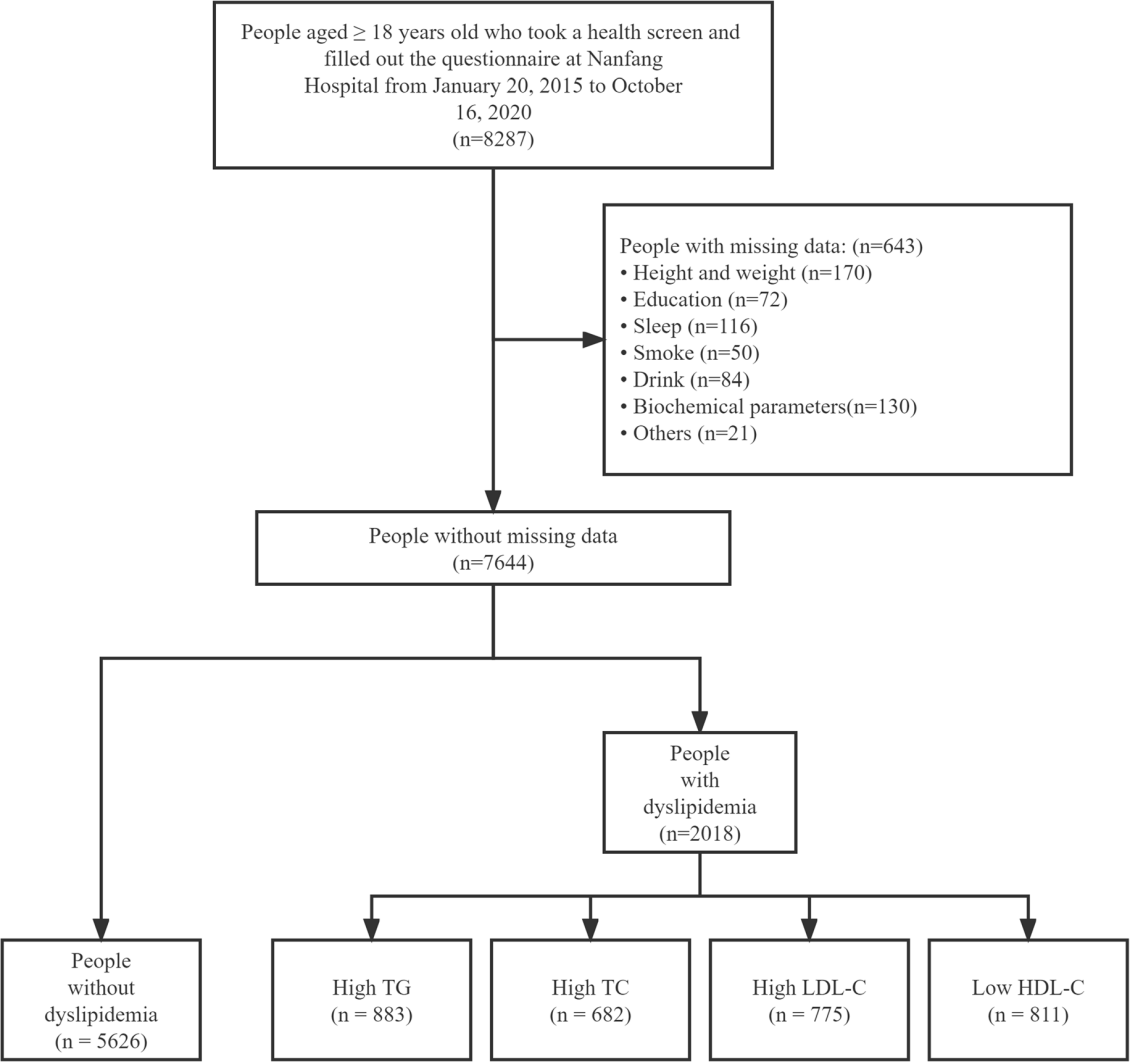
Flowchart of the patients included in the study. HDL-C: high-density lipoprotein cholesterol; LDL-C: low-density lipoprotein cholesterol; TC: total cholesterol; TG: triglycerides

**Fig. 2:**
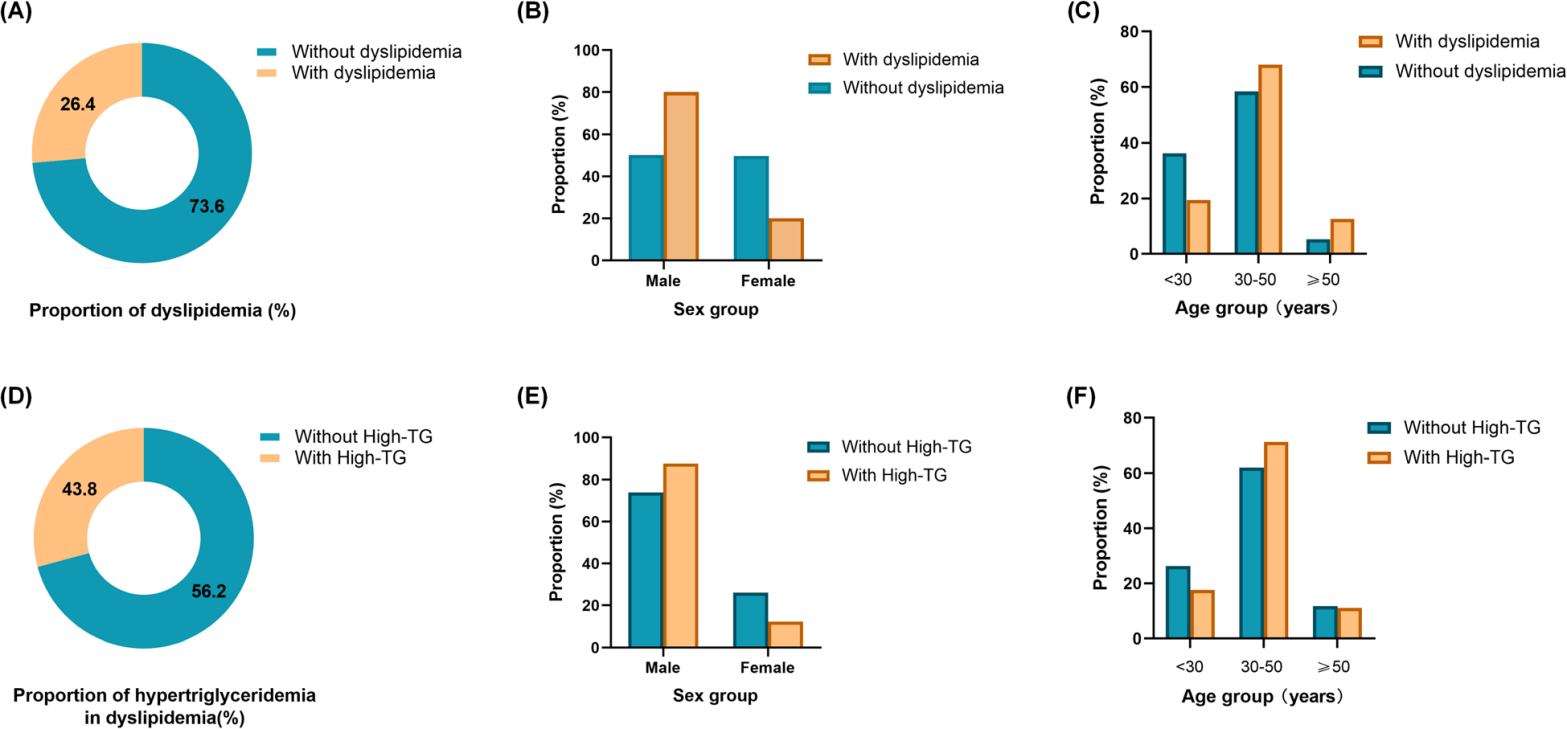
Prevalence of dyslipidemia in the population (**A**) and in different sex groups (**B**) and age groups (**C**). Prevalence of hypertriglyceridaemia in the population with dyslipidemia (**D**) and in different sex groups (**E**) and age groups (**F**). TG: triglycerides

The flow chart of this study is shown in Fig. 1. In total, 7644 people participated in this study, of whom 2018 (26.4%) had dyslipidemia, with 1614 (80.0%) being men and 404 (20.0%) being women. Table 1 shows the characteristics of participants with and without dyslipidemia. Of the total population, 61.1% were aged between 30 and 49 years. The proportion of participants who had dyslipidemia was higher among the elderly and those with BMI ≥ 24 kg/m^2^, low education, and long-term exposure to kitchen oil fumes. Compared with participants without dyslipidemia, those with dyslipidemia had higher prevalence rates of hypertension and hyperuricaemia. Compared with that of participants without dyslipidemia, the proportion of participants with dyslipidemia who were married/cohabiting was higher (77.8%) and the proportion of unmarried people with dyslipidemia was lower (19.9%). Regarding sleep, the proportion of people who sleep more than 7 h per day was lower among patients with dyslipidemia (28.0%), compared with those without dyslipidemia (30.3%). In addition, dyslipidemia was associated with business dinner attendance (*P* < 0.001). Of the participants, 770 (10.1%) were breakfast skippers while 6874 (89.9%) consumed breakfast regularly. The prevalence rates of dyslipidemia in breakfast skippers and non-breakfast skippers were 24.4% and 24.1% respectively. Figure 2 presents the prevalence of dyslipidemia in the total population and in different sex ad age groups, as well as the prevalence of hypertriglyceridaemia in participants with dyslipidemia. Table 2 shows descriptive analysis of the lipid profiles of breakfast skippers and those who consume breakfast regularly.

**Table 1:** Baseline characteristics of participants with and without dyslipidemia

Characteristics	Without dyslipidemia (*n* = 5626)	With dyslipidemia (*n* = 2018)	*p value*
**Age(y)**			< 0.001
<30	2039(36.2%)	391(19.4%)	
30–49	3294(58.5%)	1373(68.0%)	
≥ 50	293(5.2%)	254(12.6%)	
**Sex**			< 0.001
Male	2826(50.2%)	1614(80%)	
Female	2800(49.8%)	404(20%)	
**Education**			< 0.001
Under college	1031(18.3%)	454(22.5%)	
college	3192(56.7%)	1118(55.4%)	
Undergraduate and above	1403(24.9%)	446(22.1%)	
**BMI(kg/m** ^**2**^ **)**			< 0.001
<18.5	503(8.9%)	30(1.5%)	
18.5–23.9	3507(62.3%)	749(37.1%)	
≥ 24	1616(28.7%)	1239(61.4%)	
**Hypertension**			< 0.001
No	5508(97.9%)	1898(94.1%)	
Yes	118(2.1%)	120(5.9%)	
**Hyperuricemia**			< 0.001
No	3670(65.2%)	825(40.9%)	
Yes	1956(34.8%)	1193(59.1%)	
**Diabetes**			0.788
No	5613(99.8%)	2014(99.8%)	
Yes	13(0.2%)	4(0.2%)	
**Smoke**			< 0.001
Never	4585(81.5%)	1346(66.7%)	
Current	603(10.7%)	468(23.2%)	
Quit smoking for more than 6 months	157(2.8%)	75(3.7%)	
Passive smoking	281(5.0%)	129(6.4%)	
**Drink**			< 0.001
Never	2666(47.4%)	734(36.4%)	
Current	2621(46.6%)	1119(55.5%)	
Quit drinking for more than 6 months	339(6.0%)	165(8.2%)	
**Marital status**			< 0.001
Married/cohabiting	3611(64.2%)	1569(77.8%)	
Unmarried	1899(33.8%)	401(19.9%)	
Separal / divorce	97(1.7%)	43(2.1%)	
Widow	19(0.3%)	5(0.2%)	
**Long-term exposure to kitchen oil fumes**			0.036
No	5119(91%)	1804(89.4%)	
Yes	507(9%)	214(10.6%)	
**Breakfast consumption frequency**			0.075
Never	51(0.9%)	31(1.5%)	
1–3 times / week	520(9.2%)	168(8.3%)	
4–6 times / week	1755(31.2%)	633(31.4%)	
every day	3300(58.7%)	1186(58.8%)	
**Business dinner attendance**			< 0.001
Never	1111(19.7%)	325(16.1%)	
1–2 times / month	3672(65.3%)	1316(65.2%)	
1–2 times / week	706(12.5%)	312(15.5%)	
3 times / week or more	137(2.4%)	65(3.2%)	
**Daily sleep time**			0.001
< 5 h	157(2.8%)	87(4.3%)	
5–7 h	3762(66.9%)	1365(67.6%)	
>7 h	1707(30.3%)	566(28.0%)	

*BMI* body mass index

**Table 2:** Lipid profile in different group of breakfast frequency (Mean ± SD)

Breakfast frequency	TG (mmol/L)	TC (mmol/L)	HDL-C (mmol/L)	LDL-C (mmol/L)
Never	1.48 ± 0.94	5.10 ± 1.03	1.30 ± 0.26	3.31 ± 0.80
1–3 times / week	1.49 ± 1.42	4.84 ± 0.90	1.30 ± 0.26	3.07 ± 0.71
4–6 times / week	1.40 ± 1.28	4.91 ± 0.93	1.32 ± 0.28	3.13 ± 0.73
every day	1.39 ± 1.06	4.94 ± 0.94	1.33 ± 0.28	3.16 ± 0.73

*HDL-C* high-density lipoprotein cholesterol, *LDL-C* low-density lipoprotein cholesterol, *TC* total cholesterol, *TG* triglycerides, *SD* Standard deviation

### Univariate and multivariate analyses

Table 3 presents the outcome of the univariate and multivariate analyses of the risk of dyslipidemia. The univariate analysis showed that older age, male sex, higher BMI, hyperuricaemia, current smoker, alcohol consumption, and long-term exposure to kitchen oil fumes were directly correlated with dyslipidemia. Increased frequency of breakfast consumption, decreased frequency of business dinner attendance, being unmarried, having high education level, and an increased length of sleep time per day were related to a lower risk of dyslipidemia. Compared with that of those who did not eat breakfast, the OR for those who consumed breakfast daily was 0.591 (95% CI 0.377–0.928, *P* = 0.022). Higher frequency of business dinner attendance was directly related to increased dyslipidemia.

**Table 3:** Univariate and Multivariate analysis of risk factors for dyslipidemia

Characteristics	Univariate analysis			Multivariate analysis		
	OR	*95% CI*	*p* value	OR	*95% CI*	*p* value
**Age(y)**						
<30	1.000		**<0.001**	1.000		**<0.001**
30–49	2.160	1.916–2.434	**<0.001**	1.584	1.350–1.859	**<0.001**
≥ 50	4.348	3.549–5.327	**<0.001**	2.601	2.023–3.345	**<0.001**
**Sex**						
Male	1.000			1.000		
Female	0.253	0.224–0.285	**<0.001**	0.423	0.366–0.488	**<0.001**
**Education**						
Under college	1.000		**<0.001**	1.000		0.987
college	0.795	0.699–0.906	**0.001**	1.011	0.873–1.171	0.884
Undergraduate and above	0.722	0.619–0.842	**<0.001**	1.013	0.851–1.206	0.886
**BMI(kg/m**^**2**^**)**						
<18.4	1.000		**<0.001**	1.000		**<0.001**
18.4–23.9	3.584	2.459–5.224	**<0.001**	2.470	1.681–3.628	**<0.001**
≥ 24	12.855	8.829–18.718	**<0.001**	5.563	3.770–8.209	**<0.001**
**Hypertension**						
No	1.000			1.000		
Yes	2.951	2.277–3.825	**<0.001**	1.314	0.991–1.743	0.058
**Hyperuricemia**						
No	1.000			1.000		
Yes	2.713	2.444–3.012	**<0.001**	1.746	1.553–1.962	**<0.001**
**Diabetes**						
No	1.000			1.000		
Yes	0.858	0.279–2.633	0.788	0.548	0.165–1.823	0.327
**Smoke**						
Never	1.000		**<0.001**	1.000		**<0.001**
Current	2.644	2.310–3.026	**<0.001**	1.503	1.284–1.759	**<0.001**
Quit smoking for more than 6 months	1.627	1.228–2.157	**<0.001**	0.910	0.670–1.236	0.547
Passive smoking	1.564	1.259–1.943	**<0.001**	1.206	0.947–1.536	0.129
**Drink**						
Never	1.000		**<0.001**	1.000		**<0.001**
Current	1.551	1.393–1.727	**<0.001**	0.767	0.671–0.877	**<0.001**
Quit drinking for more than 6 months	1.768	1.443–2.166	**<0.001**	0.926	0.736–1.166	0.514
**Marital status**						
Married / coordinate	1.000		**<0.001**	1.000		**0.014**
Unmarried	0.486	0.430–0.550	**<0.001**	0.772	0.653–0.912	**0.002**
Separal / divorce	1.020	0.709–1.468	0.914	1.085	0.727–1.618	0.689
Widow	0.606	0.226–1.625	0.319	0.566	0.198–1.621	0.289
**Long-term exposure to kitchen oil fumes**						
No				1.000		
Yes	1.198	1.012–1.418	**0.036**	1.131	0.937–1.365	0.200
**Breakfast**						
Never	1.000		0.080	1.000		**0.001**
1–3 times / week	0.532	0.329–0.858	**0.010**	0.498	0.293–0.848	**0.010**
4–6 times / week	0.593	0.376–0.936	**0.025**	0.570	0.345–0.944	**0.029**
every day	0.591	0.377–0.928	**0.022**	0.466	0.283–0.770	**0.003**
**Business dinner attendance**						
Do not participate	1.000		**<0.001**	1.000		0.864
1–2 times / month	1.225	1.066–1.407	**0.004**	1.061	0.907–1.240	0.462
1–2 times / week	1.511	1.260–1.812	**<0.001**	1.014	0.822–1.251	0.898
3 times / week or more	1.622	1.178–2.234	**0.003**	1.061	0.740–1.520	0.749
**Daily sleep time**						
< 5 h	1.000		**0.001**	1.000		0.095
5–7 h	0.655	0.500–0.857	**0.002**	0.724	0.537–0.975	**0.033**
>7 h	0.598	0.453–0.791	**<0.001**	0.755	0.554–1.028	0.074

*BMI* body mass index, *CI* confidence intervals

In the multivariate analysis, the variables with *P* < 0.05 in the univariate analysis were included (age, sex, BMI, education, hyperuricaemia, hypertension, smoking, alcohol consumption, marital status, breakfast consumption frequency, long-term contact with kitchen oil smoke, business dinner attendance, and daily sleep time). Diabetes was also incorporated into multivariate analysis. It was found that breakfast consumption remained inversely correlated with dyslipidemia, and the OR for daily breakfast consumption was 0.466 (95% CI 0.283–0.770, *P* = 0.003).

### Adjusted variable analysis

**Fig. 3:**
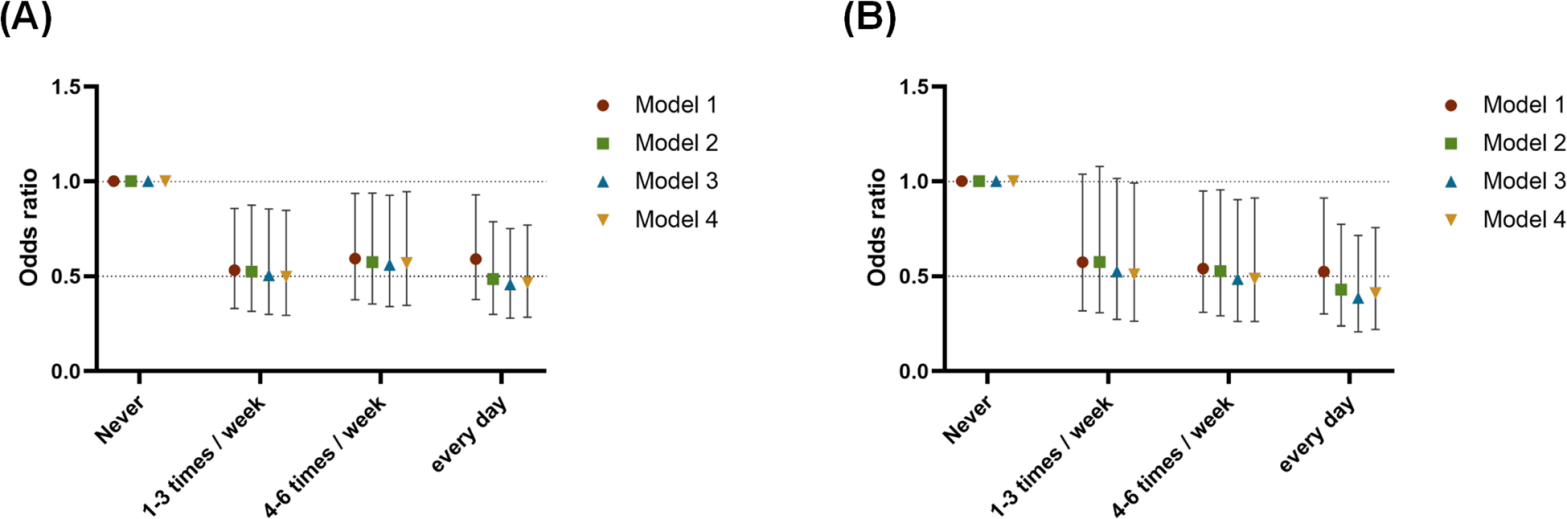
Odds ratios (95% confidence intervals) of dyslipidemia (**A**) and hypertriglyceridaemia (**B**) across breakfast consumption frequency categories. Model 1: crude model. Model 2: adjusted for age and sex. Model 3: model 2 plus adjusted for BMI, hypertension, hyperuricaemia, and diabetes. Model 4: model 3 plus adjusted for smoking, alcohol consumption, education, marital status, long-term contact kitchen oil fumes, business dinner attendance, and daily sleep time. CI: confidence interval; HDL-C: high-density lipoprotein cholesterol; LDL-C: low-density lipoprotein cholesterol; OR: odds ratio; TC: total cholesterol; TG: triglycerides

Table 4 includes different variables to analyse the OR for breakfast consumption frequency and different types of dyslipidemia. Model 1 was a crude model; Model 2 was adjusted for sex and age; Model 3 was adjusted for sex, age, BMI, hypertension, hyperuricaemia, and diabetes; and Model 4 was adjusted for smoking, alcohol consumption, education, marital status, long-term exposure to kitchen oil fumes, business dinner attendance, and daily sleep time based on Model 3. It was found that the breakfast consumption frequency was inversely related to hypertriglyceridaemia in Model 4. Figure 3 shows the ORs for dyslipidemia and hypertriglyceridaemia across breakfast consumption frequency categories in different models. In Model 4, as the breakfast consumption frequency increased, the OR became lower.

**Table 4:** Odds ratios (95% CI) of different types of dyslipidemia across breakfast consumption frequency

	Breakfast frequency	High-TG			High-TC			Low-HDL-C			High-LDL-C		
		OR	95% CI	*p* value	OR	95% CI	*p* value	OR	95% CI	*p* value	OR	95% CI	*p* value
model 1	Never	1.000		0.136	1.000		0.209	1.000		0.402	1.000		0.114
	1–3 times / week	0.574	0.317–1.037	0.066	0.487	0.248–0.955	**0.036**	0.746	0.387–1.438	0.381	0.497	0.254–0.973	**0.041**
	4–6 times / week	0.541	0.309–0.948	**0.032**	0.583	0.311–1.093	0.092	0.722	0.386–1.350	0.307	0.673	0.36–1.259	0.215
	every day	0.524	0.301–0.913	**0.022**	0.571	0.307–1.063	0.077	0.663	0.357–1.233	0.194	0.670	0.36–1.245	0.205
model 2	Never	1.000		**0.002**	1.000		**0.012**	1.000		0.119	1.000		0.058
	1–3 times / week	0.576	0.307–1.078	0.085	0.498	0.250–0.992	**0.047**	0.790	0.401–1.555	0.495	0.515	0.259–1.025	0.059
	4–6 times / week	0.527	0.291–0.954	**0.035**	0.569	0.299–1.083	0.086	0.752	0.394–1.434	0.387	0.680	0.358–1.291	0.238
	every day	0.429	0.238–0.774	**0.005**	0.452	0.239–0.856	**0.015**	0.642	0.338–1.219	0.175	0.575	0.304–1.087	0.089
model 3	Never	1.000		**0.001**	1.000		**0.01**	1.000		0.069	1.000		0.052
	1–3 times / week	0.525	0.272–1.014	0.055	0.497	0.249–0.992	**0.048**	0.761	0.382–1.517	0.438	0.516	0.259–1.029	0.060
	4–6 times / week	0.485	0.260–0.904	**0.023**	0.568	0.298–1.084	0.086	0.733	0.380–1.415	0.355	0.680	0.357–1.295	0.241
	every day	0.386	0.208–0.715	**0.002**	0.449	0.236–0.852	**0.014**	0.611	0.318–1.173	0.139	0.571	0.301–1.081	0.085
model 4	Never	1.000		**0.008**	1.000		**0.016**	1.000		0.063	1.000		0.073
	1–3 times / week	0.511	0.262–0.995	**0.048**	0.481	0.239–0.966	**0.040**	0.755	0.376–1.514	0.428	0.508	0.253–1.019	0.057
	4–6 times / week	0.487	0.260–0.912	**0.025**	0.550	0.288–1.052	0.071	0.726	0.375–1.407	0.343	0.686	0.359–1.309	0.253
	every day	0.410	0.220–0.764	**0.005**	0.442	0.232–0.841	**0.013**	0.598	0.310–1.154	0.125	0.590	0.310–1.121	0.107

*CI* confidence interval, *HDL-C* high-density lipoprotein cholesterol, *LDL-C* low-density lipoprotein cholesterol, *OR* odds ratio, *TC* total cholesterol, *TG* triglycerides

Model 1: crude model

Model 2: adjusted for age and sex

Model 3: model 2 plus adjusted for BMI, hypertension, hyperuricemia and diabetes

Model 4: model 3 plus adjusted for smoke, drink, education, marital status, long-term contact kitchen oil fumes, business dinner attendance, and daily sleep time

## Discussion

In this large study population, epidemiological evidence obtained using the retrospective cohort study design indicated that skipping breakfast was related to an elevated risk of dyslipidemia in a working population; the higher the breakfast consumption frequency, the less likely it is for triglyceride levels to be high. This result echoes the findings of many previous studies. Takebe et al. reported that, in participants aged older than 20 years, skipping breakfast was correlated with weight gain (OR 1.252, compared with a regular breakfast) [14]. A cross-sectional study in Iran showed that, among adult participants, breakfast consumption habits had a significant negative impact on the occurrence of metabolic syndrome (OR = 0.38) [13]. A South Korean study reported that consuming breakfast less frequently increases the risk of dyslipidemia [23].

In the multivariate analysis, hyperuricaemia was found to be associated with dyslipidemia. Previous studies have also reported that people with dyslipidemia have a higher prevalence of hypertension and hyperuricaemia than those without dyslipidemia [24, 25]. According to the results, dyslipidemia was more common in individuals with overweight/obesity, males, and elderly individuals, which is concordant with the outcome of previous studies [26–28]. Many studies have explored the relationship between breakfast consumption and cardiovascular disease. In a large cohort with 17–23 years of follow-up, skipping breakfast was linked to a notable increased risk of mortality from cardiovascular disease [29]. Furthermore, higher risks of cardiovascular disease, including coronary heart disease [30], stroke and haemorrhage [31], and subclinical atherosclerosis [32] were related to skipping breakfast. However, no significant correlation was found between hypertension and dyslipidemia in multivariate analysis. This may be because the information on hypertension was self-reported, and there were participants who did not realize they had high blood pressure. It has been reported that patients with diabetes have a higher prevalence of dyslipidemia, but this association was not found in the current study [33].

Univariate analysis showed that frequent social outings and long-term exposure to kitchen oil fumes were linked to a higher risk of dyslipidaemia; however, in the multivariate analysis, this significance disappeared. This study revealed that daily sleep time was related to the risk of dyslipidemia, and many previous reports have confirmed this result. People who sleep for a sufficient amount of time are less likely to have dyslipidemia than those whose sleep duration is insufficient [34]. This study showed that marital status was related to dyslipidemia. Compared with married people, unmarried people had a lower risk of having dyslipidemia; when adjustments were made for sex and age, marriage was still related to blood lipid levels. Research in this area is relatively scarce, and the relationship between marital status and dyslipidemia needs to be explained in future research.

The relationship between breakfast consumption and weight or obesity remains unclear. Some studies have shown that breakfast skipping has an insignificant role in weight gain or weight loss [35–37]. Other studies have shown that breakfast consumption frequency is related to obesity or overweight [38–40]. The type of breakfast has to be considered in determining the substantial effect on weight gain and weight loss [41]. Compared with the current study, these previous studies may have had fewer participants, and the variation in the age and sex of the population was limited.

The reasons that breakfast consumption frequency affects blood lipid levels is unclear. Previous studies have reported that skipping breakfast leads to poor daily nutrient intake, with a higher proportion of energy from fats and a higher risk of metabolic syndrome [42, 43]. The consumption of regular meals has been shown to contribute to improved body composition and metabolic status [20].

### Comparisons with other studies and what the current study add to the existing knowledge

The incidence of dyslipidemia as well as other risk factors in this Asian cohort is similar to those reported in other recent large contemporary trials [44, 45]. This research found that among the large working population, older age, obesity, male sex, hyperuricaemia, smoking, alcohol consumption, and insufficient sleep were associated with dyslipidemia. An inverse relationship between breakfast consumption and dyslipidemia was found in this Asian working population.

### Study strengths and limitations

This study has several advantages. This research explored the impact of breakfast consumption frequency on dyslipidemia in a relatively large working population in China. Many confounding factors that may have affected dyslipidemia were considered and controlled for. Previous studies have reported a relationship between lifestyle habits and blood lipid levels in general populations. The study cohort herein was composed of the working population in South China. The findings of this research may have important implications for working people’s lifestyles. An association of breakfast intake with cardiovascular disease has been reported in previous studies; thus, the results have certain guiding implications for patients who clinically need to prevent dyslipidemia.

There are some limitations to this research. First, the study used a single-centre retrospective cohort design and could not provide a reliable source of causality. Second, data on eating habits and disease history were self-reported. hence may be subjected to recall bias. Third, we did not collect data on the type of breakfast, which may have relevant effects on the lipid profile. Fourth, this study did not collect data on participants’ use of lipid-lowering drugs. Finally, due to unknown or unmeasurable factors, the influence of confounding factors could not be fully controlled.

## Conclusions

In summary, this study provided evidence that there is an inverse relationship between breakfast consumption and dyslipidemia in a working population. The higher the breakfast consumption frequency, the lower the risk of hypertriglyceridaemia. The results of this study can be used to provide suggestions on dietary habits for the working population and also to provide lifestyle guidance for patients with a clinical need to prevent dyslipidaemia.

## Supplementary information

**Additional file 1: Table S1.** Statistical results of all participants' blood lipid parameters.

## Data Availability

The datasets used and/or analysed during the current study are available from the corresponding author on reasonable request.

## Abbreviations

BMI: Body mass index
CI: Confidence interval
HDL-C: High-density lipoprotein cholesterol
HG: Hypertriglyceridaemia
LDL-C: Low-density lipoprotein cholesterol
OR: Odds ratio
TC: Total cholesterol
TG: Triglycerides
