# Supplementary material for: Breakfast consumption frequency is associated with dyslipidemia: a retrospective cohort study of a working population
Source: Lipids Health Dis. 2022 Mar 27;21:33. doi: 10.1186/s12944-022-01641-x (PMC8966363; doi:10.1186/s12944-022-01641-x)
Supplement: Supplementary file 1 — Additional file 1: Table S1. Statistical results of all participants' blood lipid parameters. [file 12944_2022_1641_MOESM1_ESM.docx]

**Supplemental table1: Statistical results of all participants' blood lipid parameters.**

| Biochemical parameters | Number (n) | Percentage (%) |
| --- | --- | --- |
| Triglyceride |  |  |
| < 2.3 mmol/L | 6761 | 88.4 |
| ≥ 2.3 mmol/L | 883 | 11.6 |
| Cholesterol |  |  |
| < 6.2 mmol/L | 6962 | 91.1 |
| ≥ 6.2 mmol/L | 682 | 8.9 |
| Low-density lipoprotein |  |  |
| < 4.1 mmol/L | 6869 | 89.9 |
| ≥ 4.1 mmol/L | 775 | 10.1 |
| High-density lipoprotein |  |  |
| ≥ 1.0 mmol/L | 6833 | 89.4 |
| < 1.0 mmol/L | 811 | 10.6 |
| Total | 7644 | 100 |
